# Some thoughts about the words we use for thinking about sex chromosome evolution

**DOI:** 10.1098/rstb.2021.0314

**Published:** 2022-05-09

**Authors:** Deborah Charlesworth

**Affiliations:** Institute of Evolutionary Biology, School of Biological Sciences, University of Edinburgh, West Mains Road, Edinburgh EH9 3LF, UK

**Keywords:** evolutionary strata, recombination suppression, master sex-determining gene, default sex

## Abstract

Sex chromosomes are familiar to most biologists since they first learned about genetics. However, research over the past 100 years has revealed that different organisms have evolved sex-determining systems independently. The differences in the ages of systems, and in how they evolved, both affect whether sex chromosomes have evolved. However, the diversity means that the terminology used tends to emphasize either the similarities or the differences, sometimes causing misunderstandings. In this article, I discuss some concepts where special care is needed with terminology. The following four terms regularly create problems: ‘sex chromosome’, ‘master sex-determining gene’, ‘evolutionary strata’ and ‘genetic degeneration’. There is no generally correct or wrong use of these words, but efforts are necessary to make clear how they are to be understood in specific situations. I briefly outline some widely accepted ideas about sex chromosomes, and then discuss these ‘problem terms’, highlighting some examples where careful use of the words helps bring to light current uncertainties and interesting questions for future work.

This article is part of the theme issue ‘Sex determination and sex chromosome evolution in land plants’.

## 1. Introduction: sex chromosomes and sex-determining loci

Problems with terminology when writing or talking about sex chromosomes are not surprising, because sex chromosomes of different organisms are at different evolutionary stages, and because our thinking about sex chromosomes is often influenced by well-studied animal systems, even though the concept of genetic sex determination and segregation of factors in heterozygous sex was first understood in plants in the very early days of genetics [1], and the first evidence for Y-linked male-determining factors came from studies in plants (reviewed in 1958 [2]), long before the mammalian SRY factor was identified [3]. New work continues to produce surprises, such as the ‘mobile sex-determining factors’ in salmonid fish [4,5] and strawberry species [6]. Great care in use of terminology is, therefore, required. Box 1 outlines an evolutionary framework, from a genome region with a new male-determining factor, defining a Y-linked ‘locus’, through to the possible eventual complete loss of this chromosome in males, to illustrate the problems and discuss some important terms (similar changes can lead to ZW systems, but, for simplicity, I will discuss XY systems). Note that, although the states diagrammed may be successive steps in the evolution of a sex chromosome pair, there is no necessary linear sequence. Indeed, the diagram shows other types of changes (discussed below), including turnover events and the loss of recombination in a physically small sex-determining region as a direct consequence of creation of a new sex-determining gene through a duplication of an autosomal gene onto the chromosome, instantly defining a new Y-linked region.

**Figure 1.**
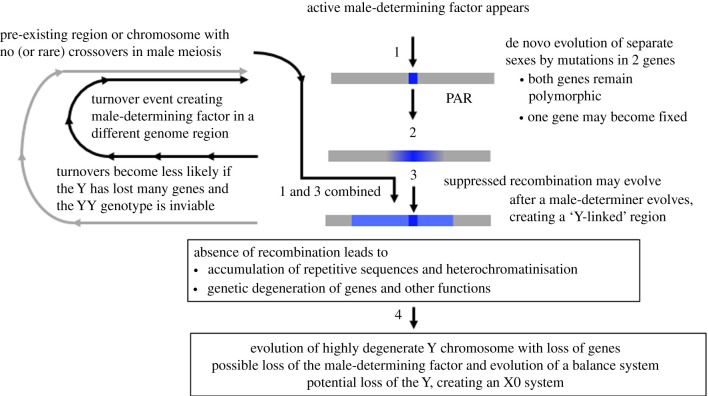
Steps in the evolution of a Y chromosome. The maleness factor is shown in dark blue, on a chromosome (coloured grey) formed mainly of partially sex-linked, or pseudo-autosomal regions, or PARs. An ‘evolutionary stratum’ is indicated by the paler blue region. If this region was non-recombining in the ancestor, before the chromosome gained the maleness factor, the entire blue region would have become Y-linked when the maleness factor arose. (Online version in colour.)

Even the most familiar terms, such as ‘sex chromosomes’, are used for very different situations in this evolutionary series. A first problem is that this term conveys the idea that the chromosome carries sex-determining genes, which is not true in species with ‘balance’ systems, including *Drosophila melanogaster* and *Caenorhabditis elegans* [7]. The *D. melanogaster* Y carries no genes with sex-determining functions [8]; sex determination involves a ‘balance’ mechanism that counts the relative numbers of X chromosomes versus autosomes, and triggers downstream pathways of male or female development [9]. This system probably evolved as a late stage in the process (labelled step 4 in figure 1 in box 1). The Y is defined as a sex chromosome based on its segregation from the X in male meiosis. It shares defining characteristics with other Y chromosomes. Notably, the lack of recombination has led to the loss of genes, through the process called ‘genetic degeneration’, which has occurred in many species' Y chromosomes, though the time-course after recombination stops is still not completely understood [10]. These changes over time can eventually allow the Y-linked male-determining factor(s) to become replaced by a balance system that no longer relies on a Y-linked male determiner.

Box 1. **Steps in Y chromosome evolution, and signs detectable in DNA sequence data.**

The figure illustrates the four main potential steps in sex chromosome evolution, and the table illustrates footprints that are detectable in DNA sequence data at different stages. Some steps, including evolution of suppressed recombination (step 3), and loss of the maleness factor, are not inevitable (see main text), and some can occur earlier or later than shown in the figure. For example, if the maleness factor arises in a non-recombining region in step 1, step 3 occurs simultaneously and can create a physically large completely Y-linked region. Conversely, turnovers can create new young systems.The steps are as follows:

— Step 1: Newly established sex-determining locus.
— Step 2: Associations develop between the locus and variants in very closely linked flanking regions. Males will be slightly more likely to be heterozygous for variants that are absent from females (indicated by lighter blue with increasing distance from the male-determining locus). Sexually antagonistic variants may accumulate in closely linked PAR genes.
— Step 3: Possible evolution of suppressed recombination, sometimes in several events. After each such event, variants arising in the region remain Y-linked; regions with male-specific variants constitute ‘strata’ of Y-X sequence divergence (see the table). The Y-linked region also starts accumulating repetitive sequences (potentially increasing the region's size) and rearrangements, potentially creating a distinctive (heteromorphic) Y chromosome. Functions start degenerating.
— Step 4: Eventual extreme genetic degeneration, deletions of genes and other sequences, possibly including the Y-linked active maleness factor (potentially greatly reducing the Y chromosome's size).

**Table 1.** Stages in Y chromosome evolution, to show the signs that are detectable in DNA sequence data from the genome region that includes the male-determining factor(s) after each of the steps shown in figure 1 has occurred.

step	male relative to female values			*F*_ST_ between the sexes in nearby genome regions
	frequency of heterozygotes	nucleotide diversity in the region	sequence coverage	
1	high if the sex-determining site arose by mutation in an existing gene (or zero if the site arose by duplication)	unaffected	1 (or 0)	unaffected
2	elevated at sites very closely linked to the sex-determining site	slightly higher than in other genome regions	1	slightly elevated
3	high across regions completely linked to the sex-determining site (strata)	high, relative to the rest of the genome	<1	higher than other genome regions
4	low (only X alleles present)	the same value in both sexes	0.5	0

Plants include groups that appear suitable for studying such evolutionary changes. For example, the genus *Rumex* includes species with X/0 sex chromosome systems, while others have XY systems [11], and the Y chromosomes of some *Rumex* species are genetically degenerated [12,13]. Flowering plants also include many taxa with variable sex systems in related species, which are suitable for studying the processes outlined in figure 1, including the interesting first step, de novo evolution of a sex-determining system from an ancestrally non-dioecious state. In angiosperms, the ancestral state can be either monoecy (in which individual plants produce both pollen-bearing staminate, or male, flowers as well as pistillate, or female, ones, so that the individual is functionally hermaphroditic), or hermaphroditism, in which individual flowers produce both stamens and pistils. Evolution from such ‘cosexual’ systems to the state in which individuals are unisexual has also, of course, occurred in animals, and changes from environmental sex determination are known [14]. However, in many animal taxa, all non-asexually reproducing species have genetic sex determination, and changes are restricted to various types of turnovers.

The de novo evolution of a sex-determining system will be discussed further below, but it is important to note here that, even when a sex chromosome does carry genes with active sex-determining functions (including ones that arise by turnover events in which a new sex-determining gene replaces a pre-existing one), problems arise because ‘sex-determining locus’ is used to mean different things. Use of the term differs between organisms, and between developmental and evolutionary biologists studying sex determination. A sex-determining ‘locus’ is sometimes called a ‘master sex-determining locus’, and defined as a genetic factor that co-segregates with the sex phenotype in families, and is completely associated with the sex phenotype in males and females from natural populations. Many aspects of sex chromosomes and sex-determination systems are shared by both plant and many animal systems, but balance systems have no such co-segregating locus. In *D. melanogaster* or *C. elegans*, the genes downstream of the ‘trigger’ have thus been termed the ‘master sex-determining locus’ [7].

## 2. Sex-determining loci and their detection using X and Y haplotypes

**Figure 2.**
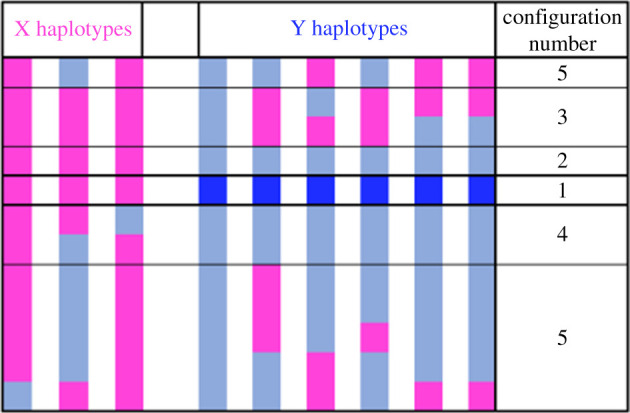
Haplotypes in a sex-linked genome, illustrating the difference between the sex-determining factor, defined as a developmental factor, and the sex-determining locus or sex-linked region, defined as a genetic locus. The columns show a small sample, of three X haplotypes and six Y ones, and the rows show the alleles present in the haplotypes at 12 variable sites, with blue indicating mutations that arose since the male-determining factor arose in the region (sites that have retained the same state in the Y and X haplotypes are not shown). At least one variant, the male-determining factor itself (dark blue, numbered configuration 1), is by definition, found only in males (and shows complete association with maleness, assuming complete penetrance and no environmental effects). This factor could be a single-nucleotide polymorphism (SNP) in a gene or a duplication into the region. New mutations in a completely Y-linked region will be male-specific (barring repeated mutation at the same site in the Y and the X), but will initially be rare among the Y haplotypes, and show partial associations with the sex phenotype; they may later become fixed in the population of Y haplotypes, and thus become completely associated with the sex phenotype. Sites with configuration number 3 have variants that are male-specific in the sample, but not fixed in the Y population, and could be either completely or partially sex linked. One site other than the male-determining factor is completely associated with the maleness factor (configuration 2), but a larger sample of females might include this variant in females, revealing that it is partially, not completely, Y-linked. Sites with configurations 4 and 5 can be diagnosed as outside the completely sex-linked region, even in the small sample diagrammed, because a variant in the Y sample also segregates in the X sample, and would be seen in females, given a large enough sample. (Online version in colour.)

Many species, however, have an active sex-determining gene or genes that defines a sex-linked region of a sex chromosome. As outlined in the next section, associations between genomic variants and individuals' sexes can then sometimes allow the sex-determining factor to be located, even if some variants are only partially male-specific, by searching sequences from natural population samples of both sexes for complete associations between the sex phenotype and sequence variants (figure 2). This approach can be used whether the sex-determining factor is a single nucleotide difference, or a ‘haplotype’ of completely associated states at many different linked sites.

The term ‘sex-determining locus’ is also used to mean just a ‘master sex-determining factor’—the gene or genes that control whether an individual will develop as a male or a female (or the major genetic sex-determining factor, if environmental effects are also important). Genes without sex-determination functions may be lost as a completely Y-linked region undergoes genetic degeneration. However, genes involved in male functions may be retained on the Y as well as the X chromosome, or (like the male-determiners themselves, in some species) may be gained by the Y by duplication of autosomal progenitors, as is true of *Drosophila* Y-linked genes [15].

## 3. Identifying sex-linked regions from linkage disequilibria

Complete sex linkage allows associations to develop between variants in DNA sequences, including male-specific variants in XY systems (figure 2). Such associations are called linkage disequilibrium (LD), and should not be confused with ‘linkage’; in a small family, variants across a large genome region may appear completely sex linked, even if the completely sex-linked region is physically small. A sample from nature, with many generations of potential crossing over in the sample's history may, however, show that much of the region is only partially sex linked. As figure 2 illustrates, all completely sex-associated variants are part of a sex-determining ‘locus’ in the genetic sense (though some or all of these variants could be partially sex linked); the locus may also include partially associated sites. It may correspond to an extensive non-recombining region, which may include many completely Y-linked genes not involved in sex determination processes, but merely in complete or strong LD with it, indicating close linkage.

A further problem is that the notation XY (or ZW) creates the idea of sex chromosome heteromorphism, but is often used for species with homomorphic pairs, which are common in plants and some other organisms with genetic sex determination, including fish. Genomic studies are providing examples in which the two members of the pair are extremely similar. In the guppy, *Poecilia reticulata* (a fish), the ‘Y’ member of the pair appears to carry the same genes at similar coverage as the ‘X’ [16], and exhaustive searches have so far discovered no consistently male-specific sequence variants, though partial associations with the male phenotype may be enriched in a ‘male-determining region’ [17,18]. These observations support other evidence that this species' Y occasionally recombines with the X [19]. Use of ‘sex chromosome’ does communicate this difference from other, completely non-recombining, Y chromosomes.

Use of ‘sex-linked region’ is preferable to ‘sex chromosome’ in species whose sex-determining locus is just a part of the chromosome, as in plants and fish with homomorphic pairs. If, however, it is a very small part, this term also requires care, to avoid obscuring the true situation. Species have been discovered with a sex-determining SNP in a recombining genome region. Variants in the surrounding region will then show at most partial associations with the sex phenotype (figure 2). Genomic analyses may fail to detect this, because the associations decay over a short physical distance. In one fish with such a sex-determining polymorphism, the tiger pufferfish, *Takifugu rubripes* (fugu), the associations are detectable only within 1 kb from the sex-determining SNP [20]; in another, *Seriola dumerili*, they decay in 20 kb, and although the ‘locus’ can be defined using these associations, it includes multiple candidate sex-determining genes [21].

At the other extreme, a male-determining gene could arise within a region where crossovers do not occur in meiosis, such as a centromeric region. The maleness factor will thereafter be completely linked to the flanking genome sequences, and new variants that arise by mutations in these ‘Y-linked’ sequences will not cross over into the corresponding X-linked haplotype and will be Y-specific (barring the unlikely occurrence of the same mutation in the corresponding site in the X haplotype). Low recombination regions can occupy large pericentromeric portions of plant chromosomes, and these ‘recombination deserts’ could account for some plant's extensive sex-linked regions [22]. Evidence for this route for the evolution of an XY pair has been found in a recent study of *Rumex hastatulus* [23].

Whenever a sex-linked region includes the chromosome's centromere, this is a possible alternative to changes in recombination that evolved under selection (see box 1 figure 1). The major selective hypothesis for closer linkage with a sex-determining locus involves a sexually antagonistic (SA) polymorphism in a partially sex-linked region, with evolution of close linkage resolving inter-sexual conflict (e.g. [24,25]). Either a mutation in a gene closely linked to the sex-determining locus establishes a polymorphism, or a new sex-determining locus arises close to an already established SA polymorphism, in a turnover event [26]. It is difficult to test for SA polymorphisms [27] or for their involvement in the evolution of non-recombining sex-linked regions. Studies of dioecious plants offer the possibility of testing whether pre-existing recombination deserts could explain why sex-determining genes are often located within non-recombining regions, without needing to invoke selection and SA polymorphism.

**Figure 3.**
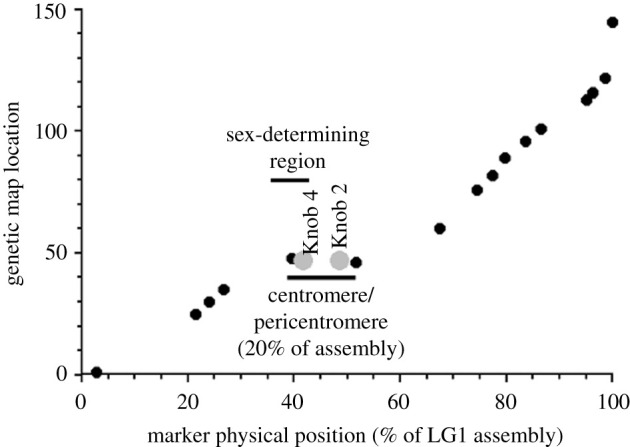
Example of a completely sex-linked region close to a plant centromeric region with a low recombination rate. Genetic map of chromosome 1 of *Carica papaya*. Large grey dots, symbolizing heterochromatic knobs, indicate the centromere position [32]. The *x* axis shows the physical positions along the chromosome, and the *y* axis shows the genetic map positions, in CentiMorgans, from published estimates for 16 genetic markers (black dots), plus the knobs [29]. The genetic map positions do not increase in the centromeric region, indicating that no crossovers occur in the region.

Current genome sequencing methods still rarely identify the centromere locations, but long-read approaches should help, as does genetic mapping combined with physical mapping from assembled genome sequences. For example, in several fish, crossovers in male meiosis are strongly localized to the chromosome ends [16,28]; the centromere locations must then correspond to the non-crossover ends. Combined genetic and physical maps are still scarce for plants, but the small Y-linked region in *Carica papaya* is near the genetic centromere location of the metacentric chromosome 1 [29–31] (figure 3), though part stopped recombining more recently [32].

## 4. Heteromorphism and homomorphism

The term heteromorphism is also not used consistently or completely clearly. It is sometimes used for species with sex chromosome-autosome fusions (such as those in *Rumex hastatulus*), a different process from heteromorphism that evolved as a consequence of loss of recombination. This contributes to uncertainty about the proportion of dioecious plants with sex chromosome heteromorphism, though the frequency of homomorphism is clearly substantial. In 1958, Westergaard's review of dioecious plants estimated very roughly that heteromorphism was detectable in around half the angiosperm species where any information was available [2]. This could be an under estimate, because some differences were probably undetectable with cytogenetic approaches used before 1958, especially when the chromosomes are small. Indeed, there is no clear criterion for heteromorphism, and the term ‘micro-heteromorphism’ may be useful for plants such as papaya, where heteromorphism was undetectable until modern cytological methods became available. On the other hand, publication biases may favour ‘interesting cases’ of plants with detectable heteromorphism.

**Figure 4.**
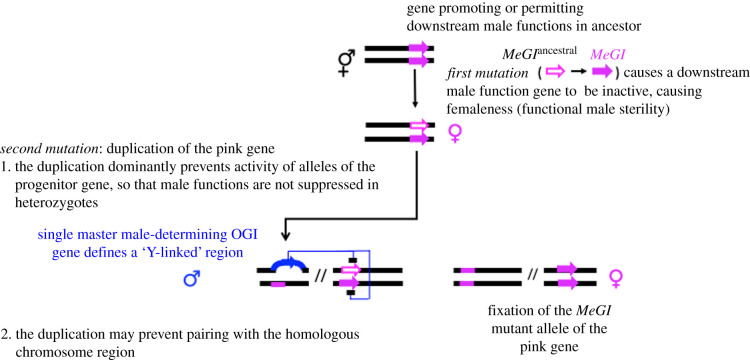
Diagram of mutations that could have produced the persimmon single gene sex-determining system [36], corresponding to one possible way in which step 1 of figure 1 could occur. The steps in the evolution of males and females are explained in the main text, and in more detail in a Supplementary text (see [22]). Note that the femaleness factor may be unlinked to the male-determining gene, and does not segregate in the population, whereas the male-determining gene segregates, and the system, therefore, shows male heterogamety. (Online version in colour.)

Homomorphism is also common among fish, in which turnover events, reviewed by Vicoso [33], appear to occur regularly. Population genomic approaches are providing evidence that some plants, like these animals (reviewed by Pan *et al.* [34]) have physically small regions of complete sex linkage. Examples include *Asparagus officinalis* [35], the persimmon [36] and *Populus tremula* [37] (see also figure 4 below). These may be young, and not yet have had enough time for the steps outlined in figure 1 to have occurred, or even for Y- or W-linked mutations to spread throughout the Y or W haplotype population; for example, some sites may have Y-specific variants found only in males, but some males may still have the same genotype as females (figure 2). Although they are challenging to study, homomorphic systems are particularly interesting, as they include systems that have undergone turnovers and sex-determining regions that have recently evolved de novo (which are of most interest for understanding how such regions first arise, as outlined below).

## 5. Evolutionary strata and genetic degeneration

Some non-recombining sex-linked regions have clearly evolved from formerly recombining regions. Sets of X-linked genes with widely different Y-X sequence divergence were first documented in humans and named ‘evolutionary strata’, whose divergence indicates the time since recombination stopped [38]. Genome regions in which variants show associations with the sex-determining locus are not the same as strata. Only the latter include completely sex-specific variants (figure 2). Partially sex-linked regions will tend to show weaker associations (though they may appear complete in small sample sizes, in which recombinant haplotypes are not included, which can also happen after bottlenecks in population size). Distinguishing between complete associations and LD in a region requires very large samples. However, if genetic degeneration is detected, this is evidence for the presence of a completely sex-linked region. If the completely sex-linked region becomes large enough, genetic degeneration will also occur, as reviewed by Bachtrog [10], and a new model suggesting a process that can act even in small completely sex-linked regions [39], which awaits empirical investigation. Completely sex-linked regions may also be detectable from chromosomal heteromorphism, with male- or female-specific inversions and deletions. However, some degree of heteromorphism may also evolve via repetitive sequencer accumulation, in the presence of rare recombination.

All these phenomena have also been found in plants. The papaya sex-determining region (see above) provides an example of evolutionary strata. Sex-linked genes present in both the X- and Y-linked regions show male-specific variants in natural populations, and Y–X divergence values differ greatly, with more diverged sequences closer to the likely centromere position [32,40]. In *Silene latifolia*, whose sex chromosome pair is highly heteromorphic, the extremely large fully sex-linked region spans the Y chromosome centromere [41,42]. Y–X divergence differences nevertheless indicate that some sequences continued to recombine (preventing divergence) long after others had stopped, though strata cannot be clearly demarcated [43–45].

Few other plants have so far been shown to have strata, and more data on Y–X or W–Z divergence are needed (ideally from synonymous sites because they evolve under low selective constraints, so that different divergence values reliably indicate different evolutionary times since divergence began, i.e. since recombination stopped). Perhaps strata rarely evolve in plants. They have not been detected in two plants with old and heteromorphic XY chromosomes. In *Cannabis sativa*, synonymous site divergence is 13–17%, and ∼30% of genes have been lost [46]. In *Coccinia grandis*, synonymous site divergence ranges up to 17%, but this appears not to represent old and young strata; most genes had very low Y–X divergence, and there appears to be only a single stratum [47,48]. Surprisingly, as many as 40 or 45% of genes are estimated to have been lost or inactivated by stop codons [47]. No strata were detected in *Mercurialis annua*, whose Y-linked region also probably evolved recently, consistent with no degeneration being detected [49].

Strata can also be studied by comparing genetic degeneration across a fully sex-linked region, as older strata should be more degenerated, and genome sequences in the region should, therefore, show lower coverage in males, relative to values in females, and to coverage of autosomal or partially sex-linked sequences, or ones in younger strata. However, coverage analyses are affected by sequence divergence values and are less reliable than synonymous site estimates using well-aligned coding regions. In a highly diverged, old stratum, it may be difficult even to determine reliably which genes are missing from the Y haplotype, especially if repetitive sequences have accumulated. Even in papaya, with physically small, well-assembled X- and Y-linked regions, gene losses have not been estimated reliably, though few genes appear to have been lost from the Y [50]. Genetic degeneration and accumulation of repetitive sequences are two consequences of suppressed recombination (another important terminological point is that evidence for the latter is not evidence that degeneration of gene functions has occurred).

## 6. Recombination suppression and master sex-determining genes

Plants are particularly valuable for studying de novo evolution of sex-linked regions in organisms that previously had no genetic sex-determining systems. The SA polymorphism hypothesis mentioned above for the evolution of Y–X suppressed recombination is closely related to the two-mutation theory for the evolution of separate sexes from an ancestrally cosexual population [51]. In both, the two mutations must be closely linked [52]. In the evolution of dioecy, the requirement for close linkage implies that non-recombining regions are the most likely to evolve sex-determining genes (and to later gain SA polymorphisms, including Y-linked maleness-enhancing factors). This requirement alone may explain (without any evolutionary changes in crossover patterns) why sex-determining loci are often within non-recombining genome regions.

Whether the ancestor had perfect flowers or was monoecious (where pre-existing genes, also termed ‘sex-determining genes’, function in suppression of male or female flower organs during development of a single individual, but are not polymorphic and cannot determine individuals' sexes), the problem of the evolution of separate sexed individuals (dioecy) is the same: how mutations can establish the genetic polymorphisms that become the ‘sex-determining factors’ and determine the (male or female) sexes of individuals. The evolutionary transition to dioecy requires at least two mutations (cosexual → female and cosexual → male). The mutations might produce flowers lacking male or female functions (sterility mutations like silkless in maize, producing wholly male plants), or, in a monoecious species, mutations might alter the proportions of male and female flowers, or male flowers of one sex express the other sex function (tassel-seed in maize is an example). Interestingly, increasing the proportion of male flowers implies lowering the female proportion, and vice versa (a form of sexual antagonism).

The two-mutation theory proposes an initial loss-of-function mutation causing male sterility (likely to be largely recessive), followed by a linked dominant SA factor with male-enhancing but female-suppressing actions. Genetic results from several plants support such a model (for example, presence of three alleles, controlling female, male and hermaphrodite sex phenotypes, in intercrosses between different races or species implies two gene differences) [2]. This is well documented in *Silene latifolia*, including from deletions demonstrating the presence of separate mutations with the above effects [2,41]. Moreover, the genetic data are inconsistent with a single-gene ‘maize trigger’ as the control of development of individuals as male or female [2], as maize geneticists had previously suggested as a route to dioecy. That model also involved implausible evolutionary changes, as the two mutations involved, tassel seed and silkless, respectively reduce male or female fertility, making the required fixation of a mutation like silkless very unlikely.

Great care is required with terminology to distinguish between the gene(s) controlling whether individuals develop as male or female in a dioecious species and those in which the mutations occurred during a transition from a non-dioecious ancestor. If one of the mutations involved in the transition becomes fixed in the species, dioecy will then be controlled solely by the different alleles at the other locus that remains polymorphic. For example, a population polymorphic for hermaphrodite (or monoecious) individuals plus females could be invaded by a mutation that specifically converts the hermaphrodites into males, without affecting the females. Importantly, such a female-suppressing allele can become fixed if it benefits the hermaphrodites, making the change invisible to genetic analysis (which relies on extant variation), though genomic and developmental analyses may reveal that it has changed in the evolution of the apparently ‘single gene’ system. As far as I am aware, no example has yet been detected.

A system with a different interaction between two genes was demonstrated in the persimmon [36]. It has been proposed [22] that this system could have evolved if the first mutation occurred in a gene (*MeGI*^ancestral^ in figure 4) that, in the cosexual ancestor, promoted both male and female functions. Female individuals (male-steriles) could then appear if the mutant allele (named *MeGI*) dominantly suppressed the maleness-promoting effect. A duplication of this gene (named *OGI*) then dominantly suppressed the mutant *MeGI* allele's expression, preventing its maleness-suppressing effect, as well as its effects permitting femaleness, thus creating males. Selection for a 1 : 1 sex ratio would then lead to fixation of the *MeGI* allele, so that presence/absence of the duplication became the sole sex-determining factor, or ‘master sex-determining gene’, and femaleness became the default sex in its absence. In this transition to dioecy, the gene in which the first mutation occurred (creating male-steriles) becomes a non-segregating autosomal downstream factor whose femaleness-permitting or promoting effects are essential for female functions when *OGI* is absent. The observation that maleness results when *MeGI* is not expressed supports the view that this allele actively suppresses male functions, and that the ancestral gene may have controlled a trade-off between male and female functions. The duplication defines a physically small sex-determining region that might be termed ‘Y-linked’ (the ‘X-linked’ region of the chromosome differs only by absence of the duplication).

Similar systems have been found in species of the *Populus* genus [37,53], which are in the Malpighiales, and distantly related to the persimmon (in the Ericales; these clades diverged from their most recent common ancestor shortly after the origin of the core-eudicots, about 120 Ma). In both cases, partial duplications of a gene important for female functions define Y-linked regions that function as the species' actively male-determining ‘trigger’ (by suppressing expression of an ARR17-like femaleness-promoting factor), and femaleness is again the default sex, in its absence. In these plants, the involvement of a duplication, coupled with knowledge about flower development, allowed the otherwise invisible *MeGI* or *ARR17*-like genes to be inferred. Note that the notations *MeGI* and ARR17-like refer to the mutant alleles, which are now fixed in these species, and that the changes from the gene's ancestral allele are not currently known in either case. However, it may be possible to infer the changes using outgroup species that have not evolved dioecy. Other systems with single gene control of male versus female sex may be discovered in the future, but the involvement of a second gene might not always be detected.

The examples just outlined demonstrate that the ‘default sex’ and ‘master sex-determining gene’ concepts cannot always be applied in plants. If both Y-linked male-sterility/fertility and male enhancing/female-suppressing alleles remain polymorphic, as in *S. latifolia*, neither is applicable; although the dominant male enhancing/female-suppressing allele is an active male-determiner, its loss leads to hermaphroditism, while loss of the other Y-linked factor produces neuter phenotypes [41,42]. These concepts are, however, applicable to the single gene sex-determining systems in persimmon and *P. tremula*. In both, the male enhancing/female-suppressing duplications act as master male-determining factors and define Y-linked genome regions, albeit physically small ones, and femaleness is the default state when this factor is absent.

Another important point is that, if only one locus remains polymorphic, no selective force exists that favours suppressed recombination in the sex-determining region (because it is the combination of two factors that causes sterility and generates selection). The ‘Y-linked’ factor in *Populus deltoides* is nevertheless within a non-recombining region, probably because the duplication arose in a non-recombining region of the species' genome [53]. Completely sex-linked regions can, therefore, be present after either route to dioecy is taken. It is also possible for such a region to evolve from a partially sex-linked region in which a SA polymorphism becomes established. A sex-linked region may thus evolve into a ‘sex chromosome’. However, this is not guaranteed, and some, perhaps most, autosomal regions that acquire sex-determining loci may never become ‘true sex chromosomes’, with extensive non-recombining regions that undergo genetic degeneration leading to low coverage of sequences in one of the sexes, and evolved sex differences in gene expression.

The models outlined here assume that dioecy evolves by major mutations that cause functional male or female sterility and become sex-determining factors. However, multiple partial female sterility mutations are probably often involved, producing male-like hermaphrodites (often called ‘inconstant males’) that can express some female functions under certain conditions [51]. The sex-linked regions in some plants may include several such factors (e.g. [35,54]). Now that it is becoming possible to identify such factors in plant sex-linked regions, it may become possible to test whether they display the increased male functions (particularly ‘trade-offs’, or sexual antagonism) that are required under this model in which males evolve gradually. If they do, the initial sterility factors should be within the oldest non-recombining region, or stratum, and other such factors might be found in younger strata, consistent with being associated with recombination suppression. In papaya, however, even though a young stratum has evolved, neither the sex chromosome as a whole, nor its fully Y-linked part, is enriched with genes whose expression in males is higher than in females [31]. However, this study found one Y-linked gene with lower expression in hermaphrodites than males, making it a good candidate for the active female-suppressing factor.

A final point is that it remains unclear whether the monoecy → dioecy transition can occur in a similar, but entirely, gradual process, without major mutations being involved that cause either male or female sterility, and whether plants will be found with single gene sex-determining systems that cannot have evolved in the way diagrammed in figure 4, but involved other types of interactions between the genes.

## Data Availability

This article has no additional data.
